# Biosensors with left ventricular assist devices

**DOI:** 10.1007/s10741-024-10413-x

**Published:** 2024-06-28

**Authors:** Mahmoud Abbassy, Muhammad Zain Ali, Riya Manas Sharma, Yohan Porus Irani, Adil Dahlan, Maimoona Azhar, Nadeem Aslam, Babar Hasan, Aamir Hameed

**Affiliations:** 1 School of Medicine, RCSI University of Medicine and Health Sciences, Dublin 2, Dublin, Ireland; 2 Internal Medicine, Kent Hospital, Brown University, Warwick, Rhode Island, USA; 3 Tissue Engineering Research Group (TERG), Department of Anatomy and Regenerative Medicine, RCSI University of Medicine and Health Sciences, 123 St. Stephen’s Green, Dublin 2, Dublin, D02 YN77 Ireland; 4 UCD School of Medicine, University College Dublin, Health Sciences Centre, Dublin 4, Belfield, Dublin, Ireland; 5 Graduate Entry Medicine, School of Medicine, RCSI University of Medicine and Health Sciences, Dublin 2, 123 St. Stephen’s Green, Dublin, D02 YN77 Ireland; 6 Division of Cardiothoracic Sciences, Sindh Institute of Urology and Transplantation (SIUT), Karachi, Pakistan; 7 Trinity Centre for Biomedical Engineering (TCBE), Trinity College Dublin (TCD), Dublin, Ireland

**Keywords:** Implantable biosensor, Left ventricular assist device (LVAD), Pressure sensor, Percutaneous biosensor

## Abstract

Heart failure imposes a significant global health burden, standing as a primary contributor to mortality. Various indicators and physiological shifts within the body may hint at distinct cardiac conditions. Specific biosensors have the capability to identify these changes. Integrating or embedding these biosensors into mechanical circulatory support devices (MCSDs), such as left ventricular assist devices (LVADs), becomes crucial for monitoring alterations in biochemical and physiological factors subsequent to an MCSD implantation. Detecting abnormal changes early in the course of disease progression will allow for improved patient outcomes and prognosis following an MCSD implantation. The aim of this review is to explore the available biosensors that may be coupled or implanted alongside LVADs to monitor biomarkers and changes in physiological parameters. Different fabrication materials for the biosensors are discussed, including their advantages and disadvantages. This review also examines the feasibility of integrating feedback control mechanisms into LVAD systems using data from the biosensors. Challenges facing this emerging technology and future directions for research and development are outlined as well. The overarching goal is to provide an overview of how implanted biosensors may improve the performance and outcomes of LVADs through continuous monitoring and closed-loop control.

**Graphical Abstract:**

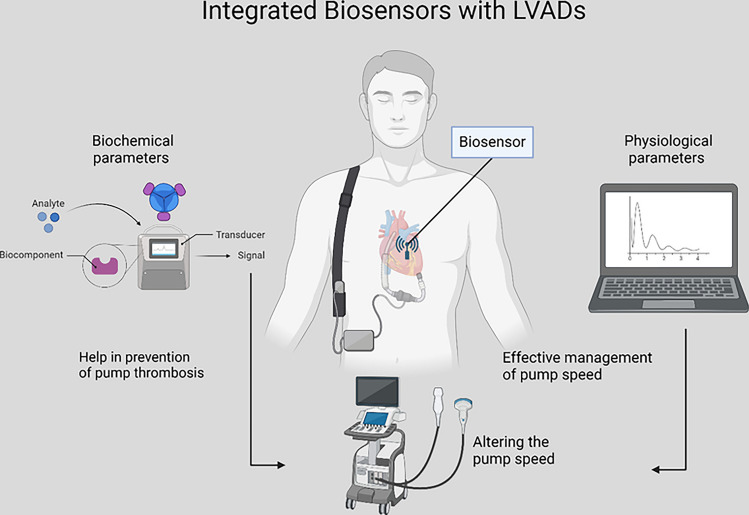

## Introduction

Heart failure (HF) is an increasingly prevalent public health concern, affecting more than 64 million people worldwide [1]. A pandemic in essence [2], HF burdens the global economy with an estimated total medical cost reaching a whooping figure of US$53.1 billion in the USA alone by 2030 [3]; and the cost of lives lost cannot be valued. Structural and/or functional cardiac abnormality leads to HF. Due to reduced cardiac output, the clinical presentation of HF includes breathlessness, fatigue and peripheral edema [4]. It is the final common manifestation of a plethora of cardiac dysfunctions and diseases. Management of HF includes a limited range of interventions, including medications that can aid in alleviating symptoms associated with HF [5]. However, the gold standard of treatment remains to be the heart transplantation. The unfortunate reality is that the demand for heart transplantation far exceeds the available supply of donor organs. This scarcity of donors demanded novel alternative interventions, such as mechanical circulatory support devices (MCSDs).

MCSDs are designed to mechanically perform or assist the pumping functions of the heart that are impaired in heart failure [6]. They can be utilised in advanced heart failure patients in two ways: as a bridge-to-transplantation to provide temporary circulatory support until a donor heart becomes available for transplantation, or as destination therapy, which is the final treatment option for patients who are not eligible for heart transplantation [4]. A wide range of MCSDs exist, including the left ventricular assist device (LVAD), the intra-aortic balloon pump (IABP), the extracorporeal membrane oxygenation (ECMO) device and total artificial heart [7]. However, the most used MCSD is the left ventricular assist device (LVAD). Between 2010 and 2020, 27,298 patients in the USA received MCSD implants. Of those, a total of 25,551 patients had LVAD implants [8]. More than 2500 LVADs are implanted annually in the USA [9].

The need for real-time information about different physiological and biochemical parameters within these patients is becoming increasingly significant for both diagnostic and prognostic purposes. Various cardiac biomarkers, including inflammatory cytokines, as well as changes in physiological measures like pressure and volume, can provide insights into different cardiac pathologies. Complications often arise following an LVAD implantation that can reduce the device’s efficacy. These complications may include infection and thrombosis, which can be detected early as an increase in the levels of specific pro-inflammatory markers [10, 11]. Early detection of such markers allows for better patient outcomes. Moreover, uncontrolled pump speed of LVADs can reduce their efficacy and can even cause dangerous arrhythmias [12]. Real-time measurement of ventricular pressure, for example, may allow for better control of pump speed.

Biosensors are analytical tools that offer such a mechanism and help detect different biochemical and physiological parameters. Coupling or implanting biosensors with MCSDs allows for real-time measurement and feedback control of the devices, ultimately improving patient outcomes. Hence, the aim of this study is to review the available biosensors that can be coupled or implanted with MCSDs, specifically LVADs, to give real-time information about different biochemical and physiological parameters. This review will also explore the different materials used to fabricate biosensors, as well as the way forward with this rapidly-developing technology.

### Cardiac cycle

The cardiac cycle refers to the mechanical events allowing the heart to pump blood sufficiently to the body. It consists of a series of pressure changes in the atria, ventricles and great vessels, which drive blood flow through the circulatory system [13]. The 2 main components of cardiac cycle are relaxation (diastole) and contraction (systole) phases. Their individual components are Systole — isovolumetric contraction, ejection, and Diastole — isovolumetric relaxation, early diastolic filling, diastasis, atrial filling [13].

The cardiac cycle begins at the end of diastole, marked by the mitral valve closure. During the initial phase of systole (isovolumetric contraction), the left ventricular (LV) pressure is still below that of the aorta, the aortic valve is closed and the ventricular volume remains unchanged. When LV pressure exceeds the pressure in the aorta, the aortic valve opens, facilitating ejection of the blood out into the aorta. As the LV contraction decreases and the LV pressure falls below that of the aorta, the aortic valve closes and that marks the end of the systole phase. The remaining volume of the blood in the ventricle at the end of the systole phase is called the end systolic volume (ESV) and is approximately 50 ml in a healthy adult [14].

As diastole begins, while the LV pressure still exceeds that of the left atrium (LA), the mitral valve remains closed and the LV volume remains unchanged; this is the isovolumetric relaxation phase. When the LV pressure falls below that of the LA (which is approximately 7 mmHg), the mitral valve opens, facilitating the blood flow from the LA to the LV. As this continues, the LA-LV pressure gradient decreases and ultimately reverses. This reversed pressure gradient decelerates and stops rapid LV blood flow in early diastole. During mid-diastole (diastasis), LV blood flow nearly stops following the equilibration of LA-LV pressure gradient. The volume towards the end of diastole, which is called the end diastolic volume (EDV) is normally 120 ml. [14]. Late in diastole, atrial contraction increases the LA pressure, introducing the LA-LV pressure gradient, promoting further filling of the LV. Post atrial systole, the atrial pressure drops with respect to the LV, and the closure of the mitral valve begins. This closure is complete with ventricular systole, which ends the diastolic phase. A similar cycle is seen on the right side as well, involving the tricuspid and pulmonary valves [13, 14].

### Heart failure

In HF, the heart is unable to sufficiently supply blood to the periphery due to a systolic or diastolic abnormality. Based on the ejection fraction (EF), which is defined as the ratio of stroke volume (SV) to EDV (EF = SV/EDV), HF is classified into:

- Heart Failure with reduced Ejection Fraction (HFrEF: EF ≤ 40%),
- Heart Failure with mid-range Ejection Fraction (HFmrEF: 41 ≤ EF ≤ 49) and
- Heart Failure with preserved Ejection Fraction (HFpEF: EF ≥ 50%).

**Fig. 1:**
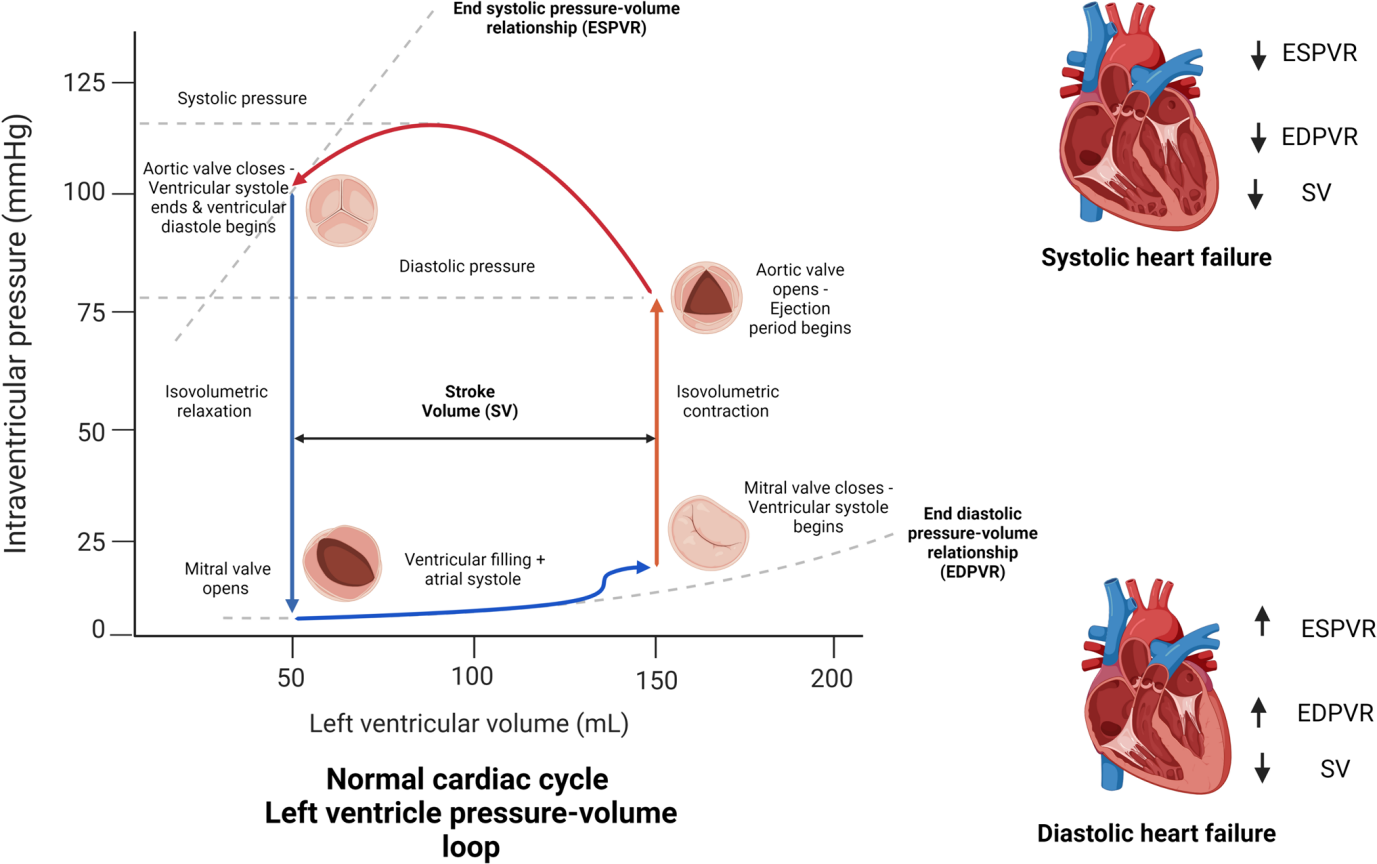
Normal cardiac cycle and derangement of some of the parameters in Heart Failure with reduced Ejection Fraction (HFrEF) and Heart Failure with preserved Ejection Fraction (HFpEF)

HFrEF or systolic heart failure, is seen in cases where SV decreases below the normal range of 50–100 ml as a result of systolic abnormality of the LV [15, 16]. The reduced contractility of the LV is initially compensated for with an increased end diastolic volume which increases the ventricular contractility — the Frank Starling mechanism. However, over a period of time, there is a reduction in stroke volume (SV) and cardiac output (CO). In HFpEF or diastolic heart failure, a diastolic dysfunction (stiff ventricle due to a cardiomyopathy for example) leads to reduced EDV resulting in a fall in SV [17]. Since, both EDV and SV are reduced, the EF remains preserved. HFmrEF sees an overlap in diastolic and systolic pathologies [15]. Figure 1 shows the normal cardiac cycle and derangement of some of the parameters in HFrEF and HFpEF.

### Left ventricular assist device (LVAD) and the need for a biosensor

A Left Ventricular Assist Device (LVAD) aids the heart in supplying the blood to the periphery/end organs. An LVAD consists of an inlet cannula in the left ventricle, from where the blood enters the pump and reaches the aorta via an outflow graft in the aorta, most commonly, the ascending aorta. Thus, an LVAD lessens the workload of the heart, reducing the increased EDV while increasing the SV to maintain the CO in a failing heart [18]. LVADs use either pulsatile or continuous flow pumps. Pulsatile pumps mimic the natural pulsatility of the cardiac cycle, while continuous flow pumps provide non-pulsatile flow. Modern LVADs primarily use continuous axial or centrifugal flow pumps, which are smaller and more durable than earlier pulsatile designs and reduce the risk of infection. LVADs are implanted via sternotomy or thoracotomy, with the left ventricular apex and ascending aorta as the standard insertion sites of the inflow and outflow cannulae respectively [11].

Despite their life-saving function, LVADs can lead to the development of complications such as right heart failure, ventricular arrhythmias, infections, and stroke. Hematological complications such as bleeding and thrombosis are common as well, with lower LVAD speeds and subtherapeutic anticoagulation increasing this risk [11, 19]. Integrating biosensors into the LVAD systems could enable real-time monitoring of parameters that provide advanced warning or help manage these complications. For example, pressure sensors could detect increasing pressures that signal impending right heart failure or stroke after an LVAD implantation. The incorporation of ECG data could help detect increasing ventricular ectopy and arrhythmias to optimise the pump speed for the patient. Sensors that analyse Von Willebrand factor activity and platelet function could assist in the development of anticoagulation regimens to optimally balance risks of clotting and bleeding in each patient. Additionally, detectors that identify elevated inflammatory cytokines could flag the immune response to an infection related to the LVAD hardware. The potential for such specific biosensors to enable individualised management of adverse events will be explored further in the following sections of this review.

## Implantable biosensors

Having a biosensor inside the body is of immense value in the diagnosis, management and treatment of patients. Implanting a biomarker-specific biosensor can reduce the ‘lag-time’ that exists due to taking serum samples of the patients, and sending them to the lab and getting the results back. Cutting down on this ‘lag-time’ would allow for a quicker response, eventually improving patient outcomes. The continuous measurements of samples in-vivo will also allow for tracking of specific trends or patterns which could be used to identify pathologies before they arise [20]. The ability to monitor patients through non-invasive means, rather than requiring constant invasive procedures like blood draws, is highly beneficial for both clinicians and patients. It provides clinicians with vital information while allowing patients greater bodily autonomy and independence from repetitive invasive interventions. Moreover, implanting pressure sensors is also of great value as it can allow for continuous monitoring of changes in pressure and volume and offer quick feedback control when necessary [21].

Both types of biosensors (for detecting biochemical parameters and physiological parameters) may yield better patient outcomes if they were to be implanted and coupled with MCSDs. Currently, there is a lack of studies on the coupling of biomarker-specific biosensors with MCSDs. The existing pre-clinical research offers means to detect cardiac biomarkers in-vitro without coupling to MCSDs. This research can be applied in the future for in-vivo testing and coupling with MCSDs. As for pressure sensors, the existing research does offer pre-clinical testing of coupling the sensor with LVADs, in both in-vitro and in-vivo settings. Moreover, studies such as the INTELLECT 2-HF study [22] in the USA and MONITOR-HF [23] in the Netherlands have investigated the use of CardioMEMS monitoring system to measure pulmonary artery pressure in patients with LVADs patients, affirming the feasibility and safety of combining pressure sensors with LVADs. Both reported a significant reduction in heart failure hospitalisations and improvement in quality of life [22, 23]. However, large prospective clinical trials on pairing pressure sensors with LVADs are yet to be conducted.

Recent advancements in developing in-vitro and in-vivo models to test new heart failure devices like biosensors and measure parameters like intracardiac pressures have provided a foundation for future clinical research to build on. For example, Andrew Malone et al. have described a mock circulatory loop that could both mimic the cardiac cycle and features two independently controlled cardiac chambers to fully simulate the blood flow and pressures of the LA and the LV [24]. Meskin et al. have recently published their work on a novel mock circulatory loop that can mimic systolic and diastolic functions of both the LA and the LV [25]. In addition to the challenges of developing large in-vivo disease models [26], considerations around animal welfare in MedTech research has further motivated the development of these in-vitro platforms. Such robust mock circulatory test beds allow for prototyping and evaluation of MCSDs while implementing the 3Rs principles — replacing, reducing, and refining the use of animals in research.

### Biosensors detecting biochemical parameters

Specific biomarkers can detect the ongoing issues with LVADs in-situ, for example, hemolysis that may lead to multiorgan failure. Timely detection of the rise in certain biomarkers may help in early intervention and prevention of any catastrophic event. Rodgers IL et al. retrospectively reviewed the levels of carboxyhemoglobin and methemoglobin in two patients with LVADs who developed significant haemolytic anemia and the levels of both the biomarkers were > 2%, indicative of the ongoing hemolysis [27].

Biomarker-specific biosensors function to detect specific target markers, such as interleukin-10 (IL-10) and tumour necrosis factor-α (TNFα). In general, they are fabricated by immobilising a specific bioreceptor onto a suitable substrate, which is placed on a transducer that recognises the interaction between the biomarker and the bioreceptor [28, 29]. The transducer is then able to convert this biochemical interaction into an electrical signal that may be processed and displayed, or trigger a feedback control mechanism, if necessary.

Two studies, both by Baraket et al. [30, 31], offered exemplary mechanisms to detect cytokines that appear due to post-LVAD implantation inflammation. Although LVADs are composed of biologically inert material, there still remains the risk of an inflammatory response at the site of the implantation of these foreign materials [30]. LVADs, particularly pulsatile flow LVADs, may lead to selective reduction in CD4 + T cells and increased apoptosis of CD4 + and CD8 + T cells. Such defects in adaptive immunity increase the risk of infection and sepsis [32]. This immune response hinders the functioning of an LVAD, reducing its efficacy, and eventually leading to worse patient outcomes.

In the first study [30], a fully integrated electrochemical biosensor was developed to detect IL-1b and IL-10 in-vitro, at minute concentrations. This biosensor was made by placing gold microelectrodes (MEs) onto a silicon substrate. The MEs were then functionalised with anti-IL-1b and anti-IL-10. This biosensor as a whole unit was then incubated in solutions with varying concentrations of IL-1b, IL-10, as well as IL-6 (just to determine the selectivity of the biosensor). The outcome of this study showed that the developed biosensor was highly sensitive for detecting the cytokines at minute concentrations ranging from 1 to 15 pg mL^−1^ [30], and selective with no interference with the other cytokines present. These findings offer an opportunity for the detection of other pro- and anti-inflammatory cytokines by using more MEs. The biosensor itself can conduct multiple measurements simultaneously, offering real-time information about many different parameters. The biosensor developed is biocompatible and could be implanted alongside LVADs. The measured cytokine levels are used to infer the inflammatory state of patients and adjust the management plan accordingly.

Another label-free biosensor developed by Baraket et al. [31] aimed to detect TNFα in patients with inflammation following LVAD implantation. The study was done in-vitro but offers mechanisms for future work to be done in-vivo. The biosensor consists of a gold surface deposited on a flexible polyimide substrate [31]. It was then functionalised by immobilising the corresponding antibodies to TNFα onto the gold electrodes. This whole biosensor unit was then placed in between the two parts of a Teflon cell to perform the electrochemical measurements. To determine the selectivity, the biosensor was exposed to different concentrations of TNFα, alongside IL-1 and IL-10. The novel biosensor developed was highly sensitive in the detection of TNFα at a range of 0.1 pg/mL to 0.5 ng/mL [31]. There was some interference as a result of non-specific binding, however, this was minimal as good selectivity was observed in the presence of other cytokines. The application of this biosensor to measure different inflammatory cytokines in plasma samples of patients after LVAD implantation is promising. Given that it is biocompatible, implanting this biosensor alongside the LVAD may yield positive patient outcomes over time.

### Biosensors detecting physiological parameters

**Fig. 2:**
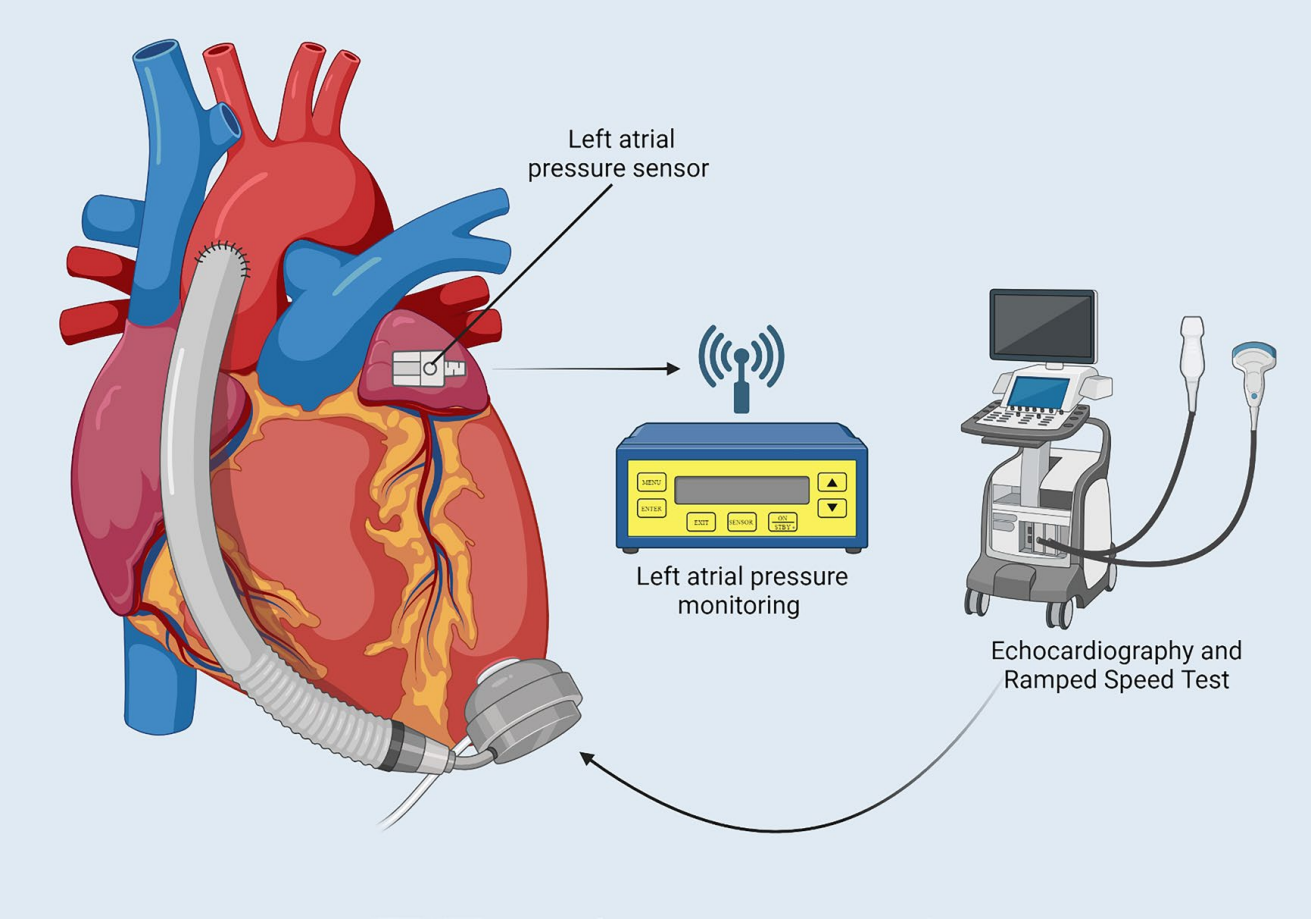
Illustration to show left atrial pressure sensor and ramped speed test

Another category of biosensors includes those designed to detect physiological parameters, such as pressure and volume (See Fig. 2 for a schematic of how they work). Changes in the blood pressure or volume following an LVAD implantation can lead to dangerous consequences, such as arrhythmias, suction events, or even reduced efficacy of the LVAD [12, 33]. As such, it is important to accurately measure the physiological changes within the body in real-time to offer quick response, and hence, better patient outcomes. Four studies reviewed here offer mechanisms to detect the physiologic parameters in patients with LVAD implantation and are tested in a variety of in-vitro, in-vivo, and human settings. Two studies also explore the potential for autonomous feedback control.

#### Left atrial pressure monitoring system

Hubbert et al. [34] describe a Titan left atrial pressure (LAP) monitoring system with an implanted Micro-Electro-Mechanical Systems (MEMS) pressure sensor. The system communicates wirelessly with an external monitor to provide a real-time LAP monitoring. Precise measurement of LAP can guide therapy to optimise the balance between pump preload and pump speed, as illustrated in Fig. 2 [34]. For this reason, the aim of this study was to report the first human application of this wireless pressure sensor in patients who are undergoing LVAD support. The Titan wireless monitoring system consists of two parts: an implantable telemetric sensor, which contains the MEMS sensor and an external monitor [34]. Four patients underwent surgery for LVAD implantation, and the LA sensor was introduced on the border between the LA and the right upper pulmonary vein. To validate the accuracy of the Titan system, simultaneous LA pressure measurements were recorded using both the wireless Titan sensor and a standard fluid-filled catheter placed in the LA for reference. The two pressure readings were compared in the first 1–2 postoperative days showing excellent correlation with only 1–3 mmHg difference between the Titan system and the fluid-filled catheter. To test the function of the Titan LAP sensor, the recordings of the four patients were monitored in the ICU as well as on the ward. Three of those patients were sent home while remotely monitoring the LAP [34]. To determine the LAP at which the LVAD pump functions at optimal speed, echocardiographic ramped speed testing was performed in each patient after discharge from the ICU and at postoperative follow-up some weeks later [34]. This study was successful in accurately measuring LAP values in patients with LVAD implantation. The pressures recorded while the three patients were discharged at home were accurately measured and the signals were sent through the internet to the hospital where a nurse would analyse them daily. The battery-free operation of this sensor also allows for long-term function without the need for maintenance or battery replacement [34]. Through echocardiographic ramped speed testing, the optimal LVAD pump speed for each patient was achieved at a LAP of 8 mmHg [34]. This indicates that daily monitoring of LAP can provide a very useful tool for assessing the pump function. With no adverse effects reported, this study demonstrates promising results for future clinical implementation [34]. Future work should include developing feedback mechanisms to allow automatic adjustment of pump speed [34].

#### Sensor to detect suction event

Fritz et al. [33] describe the development of a semi-conductor strain gage inlet pressure sensor that can detect a suction event in the inlet of a Tesla style LVAD. A Tesla style LVAD is different than other conventional pumps in that it is a shear flow pump that utilises a series of rapidly rotating discs to impart momentum in the fluid. The aim of the study was to create and design a sensor, which could monitor pressure changes throughout the cardiac cycle, and control the LVAD accordingly [33]. The sensor developed is made from titanium and the transducer is incorporated into the lumen of the titanium islet connector of the LVAD. Testing of this system included finite element analysis, sensitivity testing, drift testing, hysteresis testing, and step testing before testing the system in in-vitro and in-vivo settings [33]. In-vivo testing included a 5-h study in which the Tesla style LVAD with the inlet pressure sensor was conducted on a 105 kg calf. The inlet pressure signal from the calf was input into a custom control algorithm to automatically change the pump speed [33]. When a suction event (defined as a large drop in inlet pressure) was detected, the control algorithm instructed the pump to reduce the pump speed [33]. Drift testing revealed that over a four-week period, offset drift varied between − 180 mmHg and 140 mmHg. Unfortunately, drift of this degree precludes the use of this sensor to accurately measure physiological blood pressure [33]. Although the system could not accurately detect physiological blood pressure, it has displayed success in detecting suction events and reducing the pump speed based on the control algorithm input [33].

#### Optical biosensor coupled with an LVAD

Zhou et al. [12] describe an optical biosensor, based on the Fabry-Pérot interferometer principle, which is fabricated onto an LVAD. The Fabry-Pérot interferometer utilises two optically flat and partially reflecting surfaces arranged in parallel to form a chamber. By using a flexible diaphragm for one of the reflective surfaces, the pressure difference can be determined by the fringes generated from the optical output changes that which occur when the distance between the mirrors change [12]. The aims of this study were to develop an LVAD pressure sensor that can detect pressure changes up to 100 mmHg, respond to the changes within 10 ms, achieve a sensitivity of within 2 mmHg, be suitable for long term implantation, and not interfere with the hemodynamic environment inside the LVAD. The biosensor developed is placed on the inlet of the LVAD and detects the LV pressure using MEMS technology in patients with heart failure [12]. Parylene-C is the material used in this sensor as it is suitable for long-term implantation and is recognised as being a good structural material. The Parylene-C diaphragm is integrated directly onto the inlet shell of the LVAD and pressure change is determined by the number of fringes. The biosensor displayed success in detecting various changes in pressure within the range of 100 mmHg with a detection resolution of 1 mmHg. With larger parylene-C diaphragm sizes, the biosensor becomes more sensitive to pressure changes [12]. The sensor successfully records the pressure changes within 2 ms. This study demonstrates the development of a biosensor, which can be coupled to LVADs, to detect changes in pressure; however, further development still needs to be made in order to allow for feedback control of the LVAD. LVAD pump speed algorithms are yet to be developed that would change the speed based on the pressure signals. Ideally, both the LVAD and the biosensor power supply should be integrated. However, this remains a challenge [12].

Qawasma and Daud [35] describe an LVAD that is designed to pump blood from the LV to the aorta using an integrated Arduino Microcontroller (AMC). The aim of this study was to design a system for heart failure patients so that the LVAD pump speed could be changed based on the present physiologic status of the patient detected by an integrated ECG sensor. The ECG sensor relays its signal to the AMC through a conditional circuit and the system is designed to analyse the R-wave. The R-wave provides information about two key factors: the heart’s ability to provide the body with sufficient amount of blood and the required flow rate that needs to be achieved by the system [35]. The system adjusts the pump speed based on the time between the two successive R-waves [35]. The microcontroller analyses the number of beats and then alters the pump speed accordingly. The pump is also fitted with inflow and outflow valves, which control the direction of the blood flow [35]. A safe-mode has also been integrated into the system; so if the ECG signal is lost, or, in case of system failure, the LVAD will function at 75 beats per minute (BPM), which is predetermined in the microcontroller [35]. This system is able to respond accurately to the conditions detected from the ECG feedback. The integrated ECG successfully relays information in order to control the speed of the LVAD motor. The system accurately detects tachycardia, bradycardia and normal HR, and adjusts the flow rate accordingly [35].

## Biosensor materials

Current biosensor designs predominantly utilise miniaturised two-dimensional (2D) surfaces functionalized with immobilised biorecognition elements. It is extremely important to consider the material used for the fabrication of in-vivo biosensors. The obvious, fundamental consideration is that the device must be safe and functionally reliable. Biocompatibility, size, and flexibility are amongst other characteristics worth considering when choosing the material. Choosing the appropriate material is significant as it will directly affect the performance of the device. For example, pressure sensors are dependent on electrical conductivity to function, as such choosing a conductive material would be required. In general, the material chosen must synergise with all aspects that go into developing a biosensor: the analyte of interest, bioreceptors, substrate, as well as the transducer [36]. Some of the materials that are used include synthetic polymers, metals, carbon, glass, and silicon (Table 1). The two most commonly used materials are synthetic polymers and metals.

**Table 1:** Advantages and disadvantages of different materials used for biosensors [29, 36–40]

***Material***	***Advantages***	***Disadvantages***
***Synthetic Polymers***	• Biocompatibility • Flexibility • Low cost	• Poorer conductivity (in comparison to metals)
***Metals***	• High thermal stability • High conductivity • Good mechanical properties (tensile strength and durability)	• Expensive • Less biocompatible • Non-biodegradable
***Carbon-based***	• High conductivity • High elasticity • Low resistivity	• Non-biodegradable • Once functionalized, susceptible to contamination, reducing efficacy
***Glass/Silicon***	• Biocompatible • Flexibility • Low cost	• Non-biodegradable

### Polymers

Synthetic polymers are currently amongst the most commonly used materials for biosensor development [29]. They are the favourable choice of material for implantable cardiac biosensors as they offer great biocompatibility and ease of synthesis [29]. When choosing materials for pressure sensors, electrical conductivity is an important consideration as it provides the fundamental mechanisms for detecting changes in pressure and volume. Synthetic polymers do not offer electrical conductivity in comparison to organic polymers and metals [39]. As such, synthetic polymers would more likely be used in the fabrication of biosensors that detect specific biomarkers rather than pressure sensors. Some examples of synthetic polymers used are polyethylene terephthalate (PET), polyethylene naphthalate (PEN), and polyimide (PI).

Both PET and PEN are thermoplastic polyesters often pressed into ultra-thin films, improving their tensile strength [40]. They are used in various medical applications, apart from biosensors, such as tubing for catheters and sutures. The ability to be moulded into ultra-thin layers allows for better conformation with various biological surfaces, an advantage if they were to be implanted. Their inertness, thermal stability, and low cost gives them an advantage over using standard silicon substrates for biosensors. PEN offers slightly better characteristics in its chemical and hydrolytic resistance, thermo-oxidative resistance, and ultraviolet resistance [29]. These assets are important when considering the natural elements the biosensors will face in-vivo. A disadvantage to PEN is that it is more rigid than PET.

PI is another type of polymer that has been used in the fabrication of the Baraket et al. [31] biosensor for detecting TNFα. PI is flexible, easy to manipulate, bend, fold and mould into different shapes. It can also be deposited with metals to enhance its conductivity as it does not conduct electrical signals on its own [31]. The low cost, flexibility, high biocompatibility, and efficiency makes PI substrates an attractive choice for biosensors.

Lastly, hydrogels are three-dimensional (3D) polymers utilised in biosensors due to their mechanochemical properties. They offer advantages as an immobilisation matrix compared to 2D surfaces due to their 3D porous structure, aqueous environment, and biocompatibility. These properties increase biomolecule stability and functionality, improving biosensor sensitivity, specificity, and response time. Key biosensing parameters like target selectivity and prevention of nonspecific binding depend on the chemical composition and overall properties of the natural or synthetic polymers used to form the hydrogel. However, potential limitations, such as insufficient mechanical strength for some applications, need to be considered [36, 41].

DNA hydrogels are being used increasingly in point-of-care testing due to their cost-effectiveness and portability. Chen et al. [42] developed a DNA hydrogel biosensing platform with CRISPR/Cas14a collateral cleavage activity for detection of creatine kinase MB (CK-MB). CK-MB has been regarded as a highly specific biomarker of myocyte necrosis and elevated levels can indicate heart failure. The hydrogel contained nanomaterial signalling probes, and target binding triggered Cas14a-mediated cleavage of the DNA crosslinks, collapsing the hydrogel and releasing the nanomaterials for detection [42]. While powerful with a sensitivity of 0.355 pM, this approach still relied on traditional spectroscopic measurements for detection. Building on the versatility of DNA hydrogels, Chen et al. [43] also coupled hydrogel disruption with smartphone-based analysis for portable, quantitative visual detection. The hydrogel contained an aptamer sequence that binds to CK-MB, disrupting the crosslinks and releasing gold nanoparticles. By integrating this with a microfluidic chip, visual detection and quantification of CK-MB down to 0.027 nM was achieved using a smartphone camera [43]. Developing DNA hydrogel-based biosensors that can be implanted or attached to an LVAD system would enable real-time monitoring of relevant biomarkers such as CK-MB.

### Metals

Thin sheets made of metals such as stainless steel, titanium and copper, have also been used as substrates for pressure sensors. They offer very high thermal stability and better general mechanical properties such as tensile strength and durability in comparison to synthetic polymers. When they are pressed into thin layers, they can easily bend and provide great conductivity, giving them an advantage over other materials when fabricating pressure sensors [39]. However, producing metallic sheets is more expensive, time consuming and requires a high energy reaction, in comparison to synthetic polymers. Hence, they are not very common in the fabrication of flexible biomarker-specific biosensors [29]. Generally, metals are the appropriate choice of material only when conductivity is a necessary mechanism for the biosensor to function.

In recent years, there has been growing interest in incorporating metallic nanoparticles into biosensor interfaces due to their signal response speed and stability. Noble metal nanoparticles, particularly gold, have emerged as excellent interfacing materials for biosensors owing to their high electrical conductivity, catalytic properties, and biocompatibility [44]. Metal–organic frameworks (MOFs) have also recently emerged as promising materials for biosensing applications. MOFs are porous hybrid materials consisting of metal ions and organic bridging ligands. Compared to inorganic nanomaterials, MOFs offer potential advantages in in-vivo applications due to many reporting low cytotoxicity, inherent biodegradability, and high loading capacity [45]. Recent MOF-based composites feature higher conductivity as well through the incorporation of electroactive materials such as graphene and carbon nanotubes. Saeidi et al. [46] developed an electrochemical MOF immunosensor for the detection of cardiac troponin-I through the use of screen-printed carbon electrodes modified by gold and silver nanoparticles. Although further in-vivo testing is essential for incorporation with LVADs, this device holds considerable promise due to its remarkable sensitivity, specificity, and stability [46].

Developing implantable biosensors poses significant challenges due to the need for biocompatibility, durability, and accuracy in sensing. Ensuring seamless integration with the body's tissues while maintaining long-term functionality is a complex engineering feat. Moreover, addressing issues of power management and data transmission within the confined space of the body presents additional hurdles.

## Challenges and future work

Integrating biosensors with an LVAD poses many challenges that must be mitigated in future research. For example, the power requirement for both the biosensor and an LVAD, positioning of the biosensor, timely detection of any arrhythmia or pressure change to feedback the LVAD pump to alter its speed. How early can a sensor detect any such change? These are the challenges and the problems that have been worked upon. FIRE1, a Dublin, Ireland based Startup, for example, has developed a novel passive radio frequency-based sensor that is implanted in the inferior vena cava (IVC). This sensor allows for an early detection of volume overload as opposed to the pressure sensor by measuring the changes in the IVC cross-sectional area [47].

At present, the biocompatibility of many of these sensors when coupled onto MCSDs is yet to be determined. Biocompatible materials can mitigate this challenge. Implementing standardised protocols for biocompatibility testing is crucial. These protocols should encompass comprehensive assessments of material interactions with biological systems, such as cytotoxicity and immunogenicity tests. Another unknown of these biosensors is long-term reliability. The studies generally have shown success in their desired outcome whether detecting physiological or biochemical parameters, but the long-term reliability remains to be tested, ideally with more clinical trials. One of the studies reviewed has successfully tested the biosensor in human patients with no adverse effects reported, however, the patient population was very small, and ideally more data needs to be available at longer follow-up evaluations to accurately determine the long-term reliability [34]. One of the challenges identified with the biosensors detecting the biochemical parameters is the non-specific binding of a biomarker to the biosensor. Although this caused minimal interference during testing, the challenge remains to further maintain a minimal effect [30, 31]. Current biosensors that detect physiological parameters pose challenges relating to coupling the sensor with the integrated LVAD. Preferably, the biosensor should provide feedback to the LVAD to control the pump speed. However, as described in Zhou et al. [12], challenges remain in the development of algorithms that would change the speed based on the measured pressure. Additionally, developing reliable power supplies that would provide power simultaneously to the biosensor and the LVAD is an issue. The LVAD system described in Zhou et al. [12] does not integrate its power supply with the biosensor. Hubbert et al. [34] provide a solution to this by using a battery-free operation for the Titan pressure sensor, which implies that the power supplies do not necessarily have to be integrated. The system described in Qawasma and Daud [35] also tackles this challenge by successfully using the same battery for the ECG sensor and the LVAD. The battery level is then displayed on an integrated LCD screen, which also displays the vital signs.

Future research should include coupling of the biochemical biosensors developed directly with, or alongside the LVADs to offer real-time plasma measurements as the biosensors in these studies were not coupled [30, 31]. Additionally, as suggested in Baraket et al., more microelectrodes could be added on a single biosensor to detect multiple different biomarkers simultaneously, reducing the diagnostic ‘lag-time’ [30]. The systems described in Zhou et al. [12] and Hubbert et al. [34] can accurately detect blood pressure. However, related research in the future needs to emphasize the development of algorithms which would allow feedback control of the pump speed in response to the real-time measurements provided. In order to begin implanting these devices in patients, future research also needs to focus on testing these devices in in-vivo settings and/or clinical trials as the literature presently available predominantly offers data based on in-vitro testing, with the exception of two studies [33, 34]. Future work for the system described in Fritz et al. [33], should focus on minimising the magnitude of the drift to accurately detect the physiological pressure. If the drift can be minimized, this system provides a promising future as the algorithm has already been developed to successfully reduce the pump speed during a suction event. Additionally, refining algorithms for real-time feedback control of Medical Cyber-Physical Systems (MCPS) based on biosensor data is essential for enhancing device performance and patient safety. By integrating advanced control strategies, such as model predictive control or adaptive control, MCPS can optimise therapeutic interventions based on accurate biosensor readings [48].

## Conclusion

In conclusion, as MCSDs become more prevalent, there is tremendous potential for developing and incorporating biosensors that can provide real-time diagnostic and prognostic data. The integration of biosensors with LVADs, however, presents numerous challenges that require innovative solutions. While advancements such as the development of novel passive radio frequency-based sensors offer promising avenues for early detection of physiological changes, concerns surrounding biocompatibility and long-term reliability remain to be fully addressed. Standardised protocols for biocompatibility testing and rigorous clinical trials are imperative to ensure the safety and effectiveness of these integrated systems in real-world settings. Additionally, efforts to refine algorithms for real-time feedback control of LVADs based on biosensor data are crucial for optimising device performance and enhancing patient safety. Future research should focus on overcoming these challenges to realise the full potential of biosensor-integrated LVADs in revolutionizing cardiac care.
